# The *HIST1* Locus Escapes Reprogramming in Cloned Bovine Embryos

**DOI:** 10.1534/g3.115.026666

**Published:** 2016-03-11

**Authors:** Byungkuk Min, Sunwha Cho, Jung Sun Park, Kyuheum Jeon, Yong-Kook Kang

**Affiliations:** * Development and Differentiation Research Center, Korea Research Institute of Bioscience and Biotechnology, Daejeon, 305-806, South Korea; † Department of Functional Genomics, University of Science and Technology, Daejeon, 305-350, South Korea

**Keywords:** RNA-seq, SCNT, HIST1 cluster, reprogramming, epigenetics, HDAC inhibitor, DNA methylation

## Abstract

Epigenetic reprogramming is necessary in somatic cell nuclear transfer (SCNT) embryos in order to erase the differentiation-associated epigenetic marks of donor cells. However, such epigenetic memories often persist throughout the course of clonal development, thus decreasing cloning efficiency. Here, we explored reprogramming-refractory regions in bovine SCNT blastocyst transcriptomes. We observed that histone genes residing in the 1.5 Mb spanning the cow *HIST1* cluster were coordinately downregulated in SCNT blastocysts. In contrast, both the nonhistone genes of this cluster, and histone genes elsewhere remained unaffected. This indicated that the downregulation was specific to *HIST1* histone genes. We found that, after trichostatin A treatment, *HIST1* histone genes were derepressed, and DNA methylation at their promoters was decreased to the level of *in vitro* fertilization embryos. Therefore, our results indicate that the reduced expression of *HIST1* histone genes is a consequence of poor epigenetic reprogramming in SCNT blastocysts.

Successful cloning by somatic cell nuclear transfer (SCNT) depends on accurate reprogramming processes in which the differentiated state of the donor cell genome drifts to a totipotent, embryonic ground state (Kang *et al.* 2003). A variety of developmental defects and, consequently, low clonal viability are attributed to incomplete and poor genomic reprogramming in SCNT embryos (Amano *et al.* 2001; Eggan *et al.* 2001; Hill *et al.* 2000; Lanza *et al.* 2000; Ono *et al.* 2001). Several efforts have been dedicated to understand the physiogenetics of SCNT embryos, and to tackle the associated problem of low cloning efficiency. In this context, the most direct way to analyze SCNT embryos is to study their whole genome expression profiles. We recently performed RNA-seq in single cow blastocysts, and reported a set of differentially expressed genes (DEGs) between *in vitro* fertilization (IVF)-derived and SCNT blastocysts (Min *et al.* 2015). Additionally, we also reported features unique to SCNT blastocysts, including consistently aberrant expression of genes that function in either trophectoderm development or epigenetic modification. As an extension to the study, here we aimed to identify poorly reprogrammed regions where densely packed DEGs are coordinately regulated, and discovered a reprogramming-refractory locus. To the best of our knowledge, this is the first study to report a reprogramming-resistant megabase-sized genomic stretch in SCNT embryos.

## Materials and Methods

In this paper, we processed and analyzed preexisting cow blastocyst transcriptomes data obtained from our recent RNA-seq experiment (Min *et al.* 2015). Therefore, all raw data and related procedures, including *in vitro* fertilization and culture of bovine oocytes, somatic cell nuclear transfer, isolation and amplification of the whole transcripts, library construction for RNA-seq, differential gene expression analysis, real-time PCR validation, and Circos plot generation were described in the previous report in detail.

### In vitro fertilization of bovine oocytes

This study was carried out in strict accordance with the recommendations in the Guide for the Care and Use of Laboratory Animals of the National Livestock Research Institute of Korea. The protocol was approved by the Committee on the Ethics of Animal Experiments of the Korea Research Institute of Bioscience and Biotechnology. We obtained bovine ovaries from a local slaughterhouse (Daejon-Ojung SH, Korea), and cumulus-oocyte complexes (COC) were extracted from follicles. Oocyte maturation and IVF were performed as previously described (Park *et al.* 2007). Blastocysts at midexpanded stage were produced after 7–8 d of IVF (Kwon *et al.* 2015) and used in downstream experiments. Inseminated oocytes were *in vitro* cultured (IVC) for 20 hr in IVC medium (3 mg/ml BSA in CR1aa), and the embryos were moved to IVC medium containing 25 nM trichostatin A (TSA), and cultured for another 20 hr. Finally, the embryos were transferred back into IVC medium, and cultured to the blastocyst stage.

### Somatic cell nuclear transfer

For somatic cell nuclear transfer, bovine oocytes were enucleated, and somatic cells were injected under the zona pellucida using a micromanipulator (Leitz, Wetzlar, Germany). The injected donor and the oocyte were electrofused using an Electro Cell Manipulator 2001 (BTX). The fused eggs were activated and incubated until blastocysts were generated. Ear skin fibroblasts obtained from an adult female cow were passaged three times, and used as donor cells (Kwon *et al.* 2015). Cumulus cells obtained from COC during denudation were cultured and used as donors on the following day. We counted the cell number of each SCNT blastocyst by Hoechst staining, and picked out only healthy blastocysts comprising 60–80 blastomeres. To generate TSA-treated SCNT blastocysts, the activated oocytes were cultured in IVC medium supplemented with 25 nM TSA, and then removed to regular IVC medium 3.5 hr after. The embryos were further cultured to the blastocyst stage.

### RNA-seq of single bovine blastocysts and bioinformatic analyses

Polyadenylated RNAs were isolated from each blastocyst using Dynabeads mRNA DIRECT kit (Invitrogen). cDNAs were amplified by the pico profiling method, and Illumina sequencing libraries were constructed using the amplified dsDNAs using TruSeq DNA Sample Prep kit (Illumina). For bioinformatic analyses, TopHat-HTSeq-DESeq pipeline was followed. Raw read data from HiSeq2500 were aligned on *Bos taurus* UMD3.1 using TopHat, and gene expression levels were calculated using HTSeq followed by DESeq.

### qPCR validation of HIST1 histone gene expression

For quantitative real-time PCR (qPCR) validation of *HIST1* histone gene expression, cDNAs were prepared from six male IVF or fSCNT blastocysts. One-twentieth volume of cDNA was mixed with 10 μl TOPreal qPCR 2X PreMIX (Enzynomics), and 10 μM of *HIST1* histone gene-specific primers. Quantitative PCR was performed in a 7500 Real-Time PCR System (Applied Biosystems). All experiments were performed in triplicate. The list of PCR primers is as follows: AACAAGCTGCTGGGCAAAGT and CGTTGTTTCCAATCTTGGTTC for *HIST1H2AJ*; CAAGCTGCTGGGTAAAGTCA and CGAAGTAATCCAGACTTCTA for *HIST1H2AG*; GCAAAGTCACCATCGCTCAG and CACTGAGATCTAGGTAGGTTCA for LOC618824.

### MSRE-PCR

Genomic DNAs were extracted from 20 IVF or fSCNT blastocysts. For this, blastocysts were incubated in embryo lysis buffer (50 mM KCl, 2.5 mM MgCl_2_, 0.1 μg/ml gelatin, 0.45% NP40, 0.45% Tween20, 10 mM Tris-Cl, and 200 μg/ml proteinase K) at 55° for 1 hr followed by heat inactivation of proteinase K at 85° for 10 min. Whole lysates were reacted with 10 U of *Hpa*II (NEB) or *Msp*I (NEB) at 37° overnight in 40 μl reaction volume (Yeo *et al.* 2007). Forty cycles of PCR was carried out on 1 μl digestion products using *HIST1* histone-promoter-specific primers: TCAGAGCCTGCCAGCCAGG and AACTCGGGTACAAGTGGCAA for *HIST1H1C*; CACAGAGCAGGCAACCAATCATCA and TGCTTCTGTTTAGCCAGGAAAGA for *HIST1H1D*; AAGAAGAAGGCGAAGAAATCGG and CAGCCAGAGACACGCCACT for *HIST1H1E*; TTCACGAACGAATTCATGATGCCC and GAAGAAGGACGGCAAGAAGC for *HIST1H2AG*; GAGCTACTCCGTGTACGTGTA and AAATGTCGTTGACGAAAGAGTTCATGA for *HIST1H2B*; TTCACGAACGAATTCATGATGCCC and GCAGAAGAAGGACGGCAAGAA for *HIST1H2BB*; TACTTGGAGCTGGTGTACTTGG and AAGCGCTCGACCATCACATCTAG for *HIST1H2BD*; and ATTACAACAAGCGCTCGACCAT and GTTGCTGGACAACTTTACTTGG for *HIST1H2BN*.

### Data availability

Global expression data used in this study is available in our previous report (Min *et al.* 2015)

## Results

**Figure 1:**
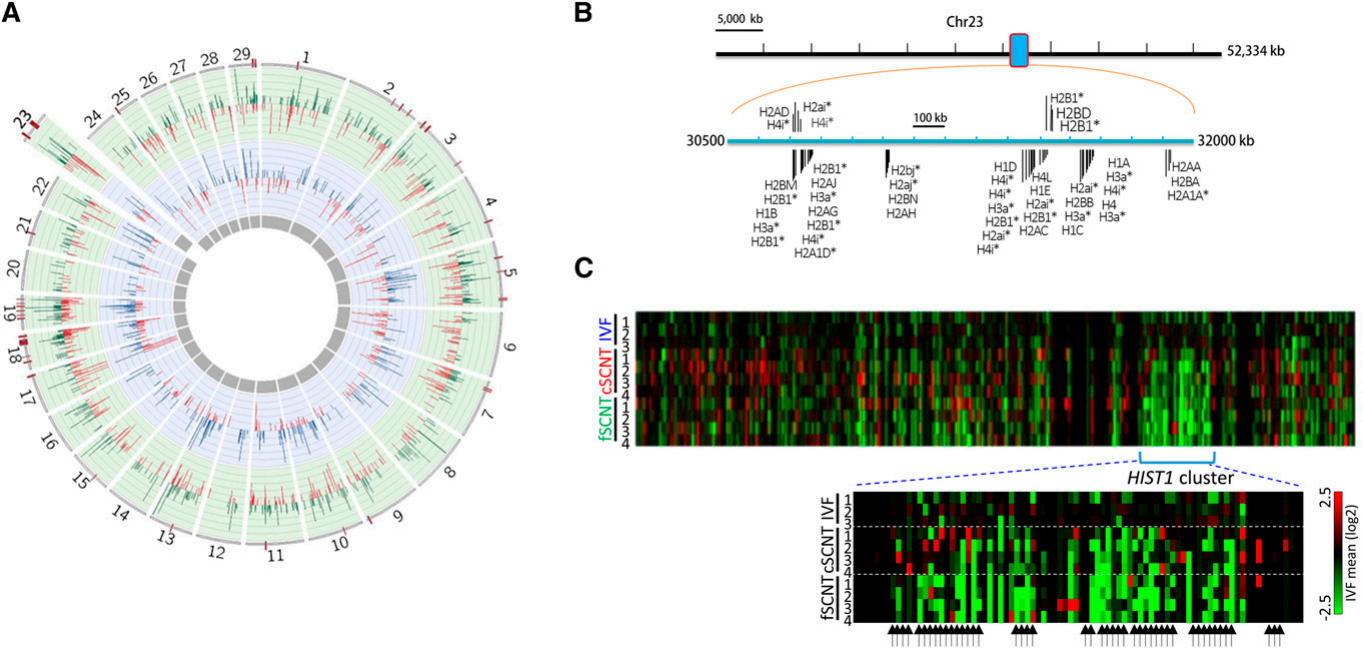
The *HIST1* cluster is downregulated in somatic cell nuclear transfer (SCNT) blastocysts. (A) Circos plot representing significant differentially expressed genes (DEGs; *P* < 0.05) in cumulus-cell (cSCNT; blue, inner circular layer), and fibroblast SCNT blastocysts (fSCNT; green, outer circular layer), *vs.* those in *in vitro* fertilization (IVF) blastocysts. Their fold-change values are shown (blue, green, and red bars within the circular layers). Red bars on the outermost layer represent DEG-rich regions in cSCNT, and/or fSCNT (five or more DEGs per 2 Mb window). Numbers on the outer layer indicate chromosome numbers; X and Y chromosomes are omitted. (B) Distribution of histone genes within the *HIST1* cluster, ranging from 30.5 Mb to 32 Mb, on chromosome 23. Predicted histone genes are marked by asterisks. Only histone-related genes are shown, and other genes in the *HIST1* locus are omitted. (C) Heat map of genes in the *HIST1* cluster showing relative gene expression of single blastocysts to the mean levels of IVF group along the length of chromosome 23. Below, the enlarged *HIST1* cluster. Arrows indicate annotated histone genes including predicted ones.

We recently reported RNA-seq data from single bovine blastocysts obtained by either IVF or SCNT. For the latter, we used two different donors, cumulus cells (cSCNT) and ear-skin fibroblasts (fSCNT) (Min *et al.* 2015). We collected an average of 12.7 million high-quality paired-end reads, and generated an expression profile dataset using TopHat and HTSeq, and *Bos taurus* UMD3.1 as reference genome. Analysis using DESeq revealed 630 and 1064 DEGs in cSCNT and fSCNT blastocysts, respectively, *vs.* IVF blastocysts. To identify poorly reprogrammed genomic regions where coordinately regulated DEGs are densely packed, we scanned the entire genome of SCNT blastocysts for erroneous domains by the sliding window analysis. Thirty-eight windows were detected to have six or more DEGs in single windows in the fSCNT blastocysts (Figure 1A). Of these, 13 were common to cSCNT blastocysts.

Among the DEG-rich loci, we were particularly interested in a locus where histone genes are bundled together. In both human (Albig *et al.* 1997) and mouse (Wang *et al.* 1996), about 50 histone genes heavily populate this locus, which is called the *HIST1* cluster, and are transcriptionally regulated in a coordinated manner (Marzluff *et al.* 2002; Marzluff and Duronio 2002). The bovine *HIST1* cluster on chromosome 23 resembles those of human and mouse. It spans about 1.5 Mb, and contains 47 histone and histone-like genes on *Bos taurus* UMD3.1 (Figure 1B). A heat map of genes on chromosome 23 unambiguously pinpointed downregulation at the *HIST1* cluster in both cSCNT and fSCNT blastocysts (Figure 1C). When the expression of HIST1 histone genes was examined in donor fibroblasts, SCNT blastocysts appeared, overall, to be closer to donor cells than IVF blastocysts (Supplemental Material, Figure S1).

**Figure 2:**
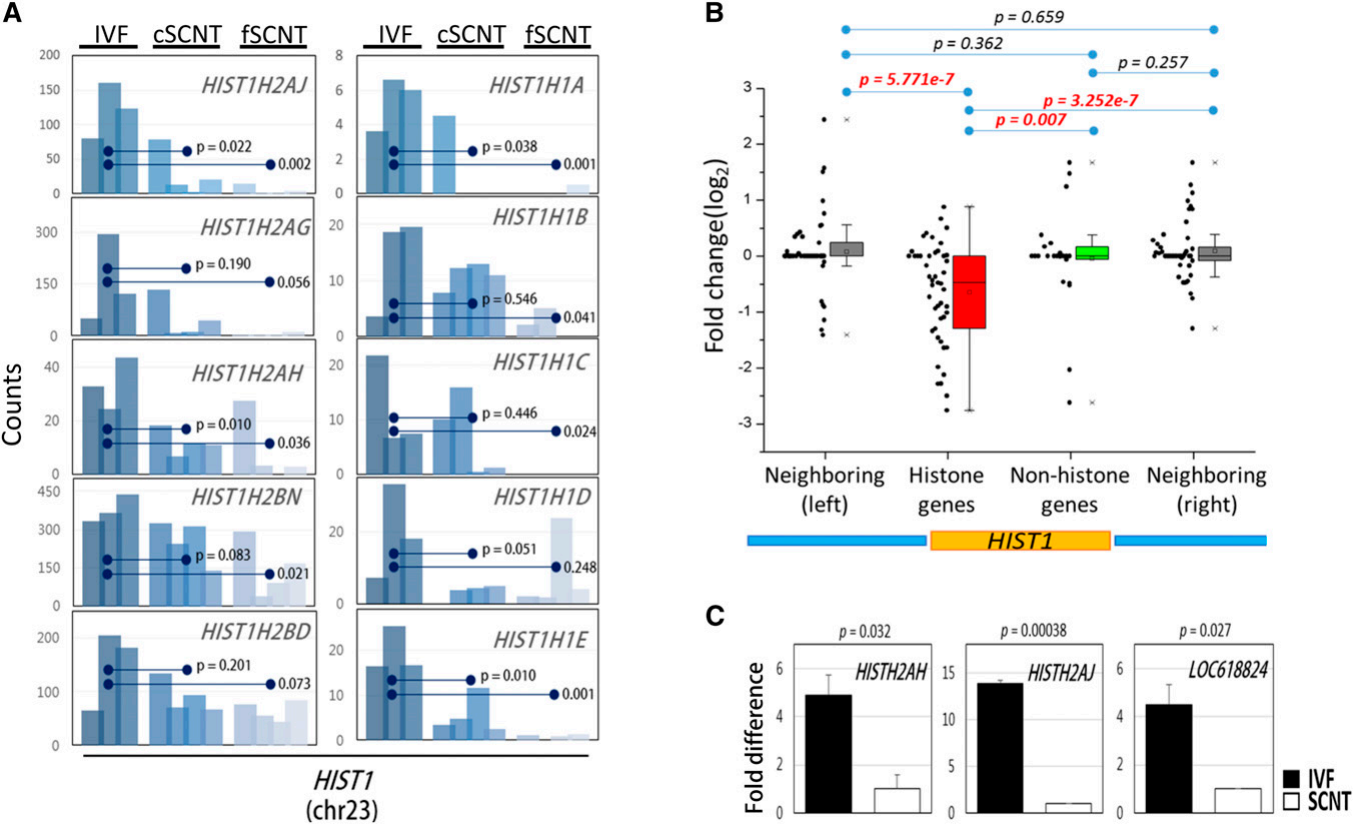
Downregulation of histone genes at the *HIST1* cluster in somatic cell nuclear transfer (SCNT) blastocysts. (A) Expression profiles of *HIST1* histone genes in single blastocysts. Bar graphs represent the expression of representative *HIST1* histone genes in individual blastocysts between cSCNT or fSCNT groups *vs.* IVF embryos. Statistical significance between the groups is denoted (Student t-test). (B) Box plot showing relative expression of *HIST1* histone genes in both cSCNT and fSCNT groups *vs.* those in the IVF group. For comparison, 47 histone and 26 nonhistone genes within the *HIST1* cluster (yellow box below), as well as the 57 (left) and 47 (right) upstream and downstream genes, respectively (blue boxes), are included. (C) Validation of the *HIST1* histone genes in IVF (solid bar) and fSCNT (blank bar) blastocysts by qPCR. Error bars indicate standard deviation of replicated experiments.

Detailed inspection of the transcriptomes revealed that individual histone genes residing in this cluster were, indeed, weakly expressed in both the fSCNT and cSCNT groups (Figure 2A). We further examined the expression of 47 *HIST1* histone genes independently of 26 nonhistone genes in the same cluster. We also examined 47 and 57 genes upstream and downstream of the cluster, respectively (Figure 2B). Interestingly, only the *HIST1* histone genes were significantly downregulated in SCNT blastocysts, whereas genes of other subsets, including the nonhistone genes within the *HIST1* cluster, were not altered. This result agrees with the previous finding that histone genes in the human *HIST1* cluster are coordinately regulated (Marzluff and Duronio 2002). By qPCR using cDNAs from freshly prepared IVF and fSCNT blastocysts, we confirmed that the *HIST1H2AJ*, *HIST1H2AH*, and *LOC618824* (*H2AI*-like) genes in the *HIST1* cluster were significantly downregulated in the SCNT blastocysts (Figure 2C). Furthermore, we found that bovine SCNT blastocysts also expressed genes, such as *YY1*, *POU2F1*, *NPAT*, and *SLBP*, known to be involved in coordinated regulation of *HIST1* histone genes in human and mouse (Eliassen *et al.* 1998; Fletcher *et al.* 1987; Ma *et al.* 2000; Zhao *et al.* 2000; Whitfield *et al.* 2000). However, their expression in SCNT blastocysts was comparable (*NPAT* and *SLBP*) to, or even higher (*YY1* and *POU2F1*) than, that in IVF blastocysts (Figure S2).

**Figure 3:**
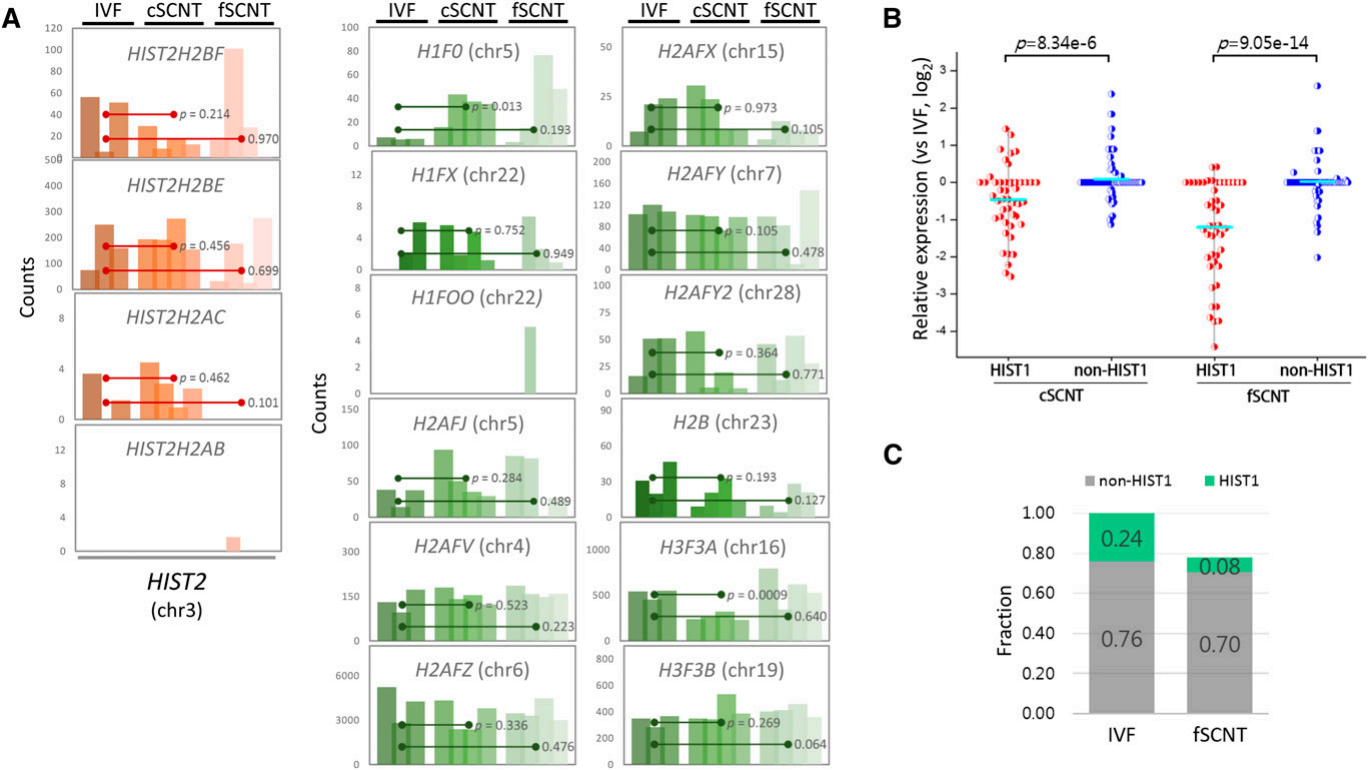
Non*HIST1* histone gene expression in SCNT blastocysts. (A) Expression profiles of non*HIST1* histone genes including *HIST2* histone genes in individual blastocysts. Statistical significance between the groups is denoted (Student t-test). Chromosome location of each histone gene is indicated in parenthesis. (B) Relative expression of *HIST1* (red dots) and non*HIST1* (blue dots) histone genes in cSCNT and fSCNT groups *vs.* the IVF group. Mean expression levels are shown as cyan-colored bars. The statistical significance between *HIST1* and non*HIST1* histone gene groups are indicated. (C) Comparison of expression levels of *HIST1* and non*HIST1* histone gene groups between IVF and fSCNT blastocysts. Green and gray colors represent the fractions of *HIST1* and non*HIST1* histone genes expressed, respectively.

In contrary to *HIST1* histones, histone genes such as *H2AFV*, *H2AFY*, and *H2AFZ* on other chromosomes (4, 7, and 6, respectively), and those in the *HIST2*-like cluster on chromosome 3 (*HIST2H2AB*, *HIST2H2AC*, *HIST2H2BE*, and *HIST2H2BF*) showed no difference in expression between the blastocyst groups (Figure 3A). When the *HIST1* histone genes were compared with ∼100 other histone genes residing elsewhere, their expression levels differed significantly between cSCNT and fSCNT (*P* = 8.34e–6, and 9.05e–14, respectively; Figure 3B). The sum of expression of all the histone and histone-like genes in the genome revealed that the fSCNT group expressed them at about 80% of what the IVF group did (Figure 3C). Notably, while the *HIST1* histone gene expression took ∼24% out of total histone gene expression in the IVF group, their contribution was only 8% in the fSCNT group, which was one-third (0.08/0.24) of the former. This indicates that the difference in total histone gene expression between the IVF and SCNT groups is derived largely from the difference in expression of the *HIST1* histone genes.

**Figure 4:**
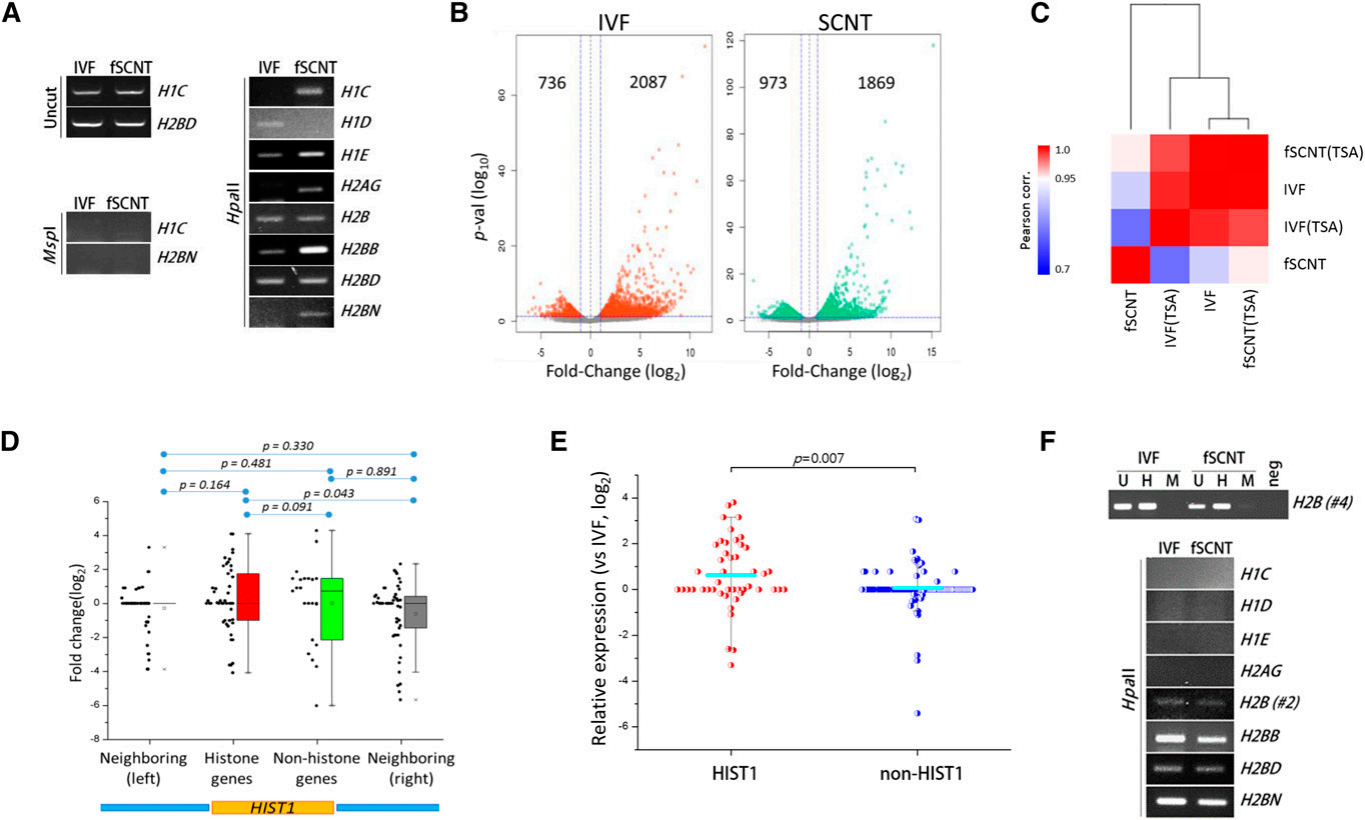
Epigenetic regulation of *HIST1* histone gene expression. (A) MSRE-PCR for analysis of DNA methylation at *HIST1* histone gene promoters. Genomic DNAs obtained from blastocysts (*n* = 10) of each group were first digested with either *Hpa*II or *Msp*I. This was followed by PCR using gene-specific primers. Both enzymes equally recognize the 5′-CCGG-3′ sequence, but *Hpa*II cannot function when the second cytosine is methylated. Stronger band intensity in the gel electrophoresis image indicates higher methylation level at the corresponding promoter. PCR was repeated twice for each gene promoter. Uncut, positive control; *Msp*I, negative control. (B) Trichostatin A (TSA)-mediated global gene expression increase in IVF and fSCNT blastocysts. Differentially expressed genes (DEGs; *P* < 0.05, fold-change > 2) in TSA-treated blastocysts *vs.* the corresponding untreated groups are shown in either red or green. In TSA-treated IVF and fSCNT blastocysts, 2087 and 1869 genes were upregulated, and 736 and 973 downregulated, respectively. Horizontal blue line indicates *P* = 0.05, while vertical ones indicate log_2_(fold change) = ± 1. (C) Heatmap for Pearson correlation between TSA-treated or untreated IVF and fSCNT blastocyst groups. (D) Expressions of *HIST1* histone genes in SCNT blastocysts relative to those in IVF blastocysts treated with TSA. The same subsets of genes in Figure 2B were assessed. (E) Expressions of *HIST1* (red) and non*HIST1* (blue) histone genes in TSA-treated fSCNT relative to that in TSA-treated IVF blastocysts. Cyan bars indicate mean expression of either *HIST1* or non*HIST1* histone genes. Student’s *t*-test was performed to determine statistical significance. (F) MSRE-PCR analysis of DNA methylation at *HIST1* histone gene promoters in TSA-treated IVF and fSCNT blastocysts. Upper panel, control experiment; H, *Hpa*II digested gDNA; U, uncut gDNA for positive control; M, *Msp*I digested gDNA for negative control. Two different promoter regions, *H2B* #2 and #4, were analyzed for *H2B*, and the latter is represented as a control experiment.

Given the possibility that the *HIST1* cluster is epigenetically regulated, we examined the epigenetic state of this region. First, DNA methylation at various *HIST1* histone gene promoters was analyzed by methylation-sensitive restriction enzyme polymerase chain reaction (MSRE-PCR), using *Hpa*II-digested genomic DNA from either IVF or fSCNT blastocysts as template. We found that most of the promoters (*HIST1H1C*, *HIST1H1E*, *HIST1H2AG*, *HIST1H2BB*, and *HIST1H2BN*) examined were more heavily methylated in the SCNT samples, whereas the rest were either similarly (*HIST1H2B* and *HIST1H2BD*) or less (*HIST1H1D*) methylated (Figure 4A). The results suggest that, in general, gene promoters at the *HIST1* locus retain aberrant DNA methylation states in SCNT blastocysts. Next, we addressed whether this abnormal epigenetic state of the *HIST1* cluster could be relieved by treatment with TSA, a histone deacetylase (HDAC) inhibitor. TSA positively influences animal cloning by promoting chromatin relaxation and increased global gene expression (Kishigami *et al.* 2006, 2007; Jee *et al.* 2012; Cui *et al.* 2011). RNA-seq was performed in TSA-treated IVF and fSCNT blastocysts. Differential expression analysis showed that TSA treatment upregulates a large number of genes in both blastocyst groups, as expected (Figure 4B). Pearson correlation analysis showed that correlation coefficient was the highest (*r* = 0.998) between IVF and TSA-treated fSCNT groups, which was a substantial increase considering the relatively low coefficient (*r* = 0.911) between them in the absence of TSA (Figure 4C). Examination of the genes in the *HIST1* cluster revealed that, in contrast to the results shown in Figure 2B, the expression of histone and nonhistone genes within the *HIST1* cluster did not significantly differ at *P* < 0.05 (Figure 4D). Compared with non*HIST1* histone genes, contrary to the result in Figure 2B, *HIST1* histones showed increased expression in TSA-treated SCNT blastocysts (Figure 4E). Finally, we analyzed TSA-treated SCNT blastocysts for methylation state using MSRE-PCR. We observed that the methylation states of both SCNT and IVF blastocysts looked similar to each other at the *HIST1* histone gene promoters after TSA treatment, suggesting that TSA might directly or indirectly induce demethylation of the *HIST1* histone gene promoters (Figure 4F).

## Discussion

We found that histone genes in the *HIST1* cluster were coordinately and epigenetically downregulated in SCNT blastocysts. Our findings suggest that, compared with IVF embryos, SCNT embryos have more repressive chromatin in the *HIST1* locus, which hinders transcription factors approaching the region. Histone genes in the human and mouse *HIST1* loci are known to be coregulated by YY1, POU2F1, NPAT, and SLBP, but these genes are found normally expressed in cow SCNT blastocysts (Figure S2). This implies that the presence of these regulators is not enough to derepress the *HIST1* locus, and that the *HIST1* region exists in a transcriptionally nonpermissive chromatin state that is highly resistant to reprogramming in SCNT embryos. This is supported by heavier methylation at the *HIST1* histone gene promoters in SCNT (Figure 4A). The low expression of *HIST1* histone genes in donor cells (Figure S1) leads to an assumption that the *HIST1* histone gene expression anomaly shown in SCNT embryos might have originated in donor cell genomes. Figure 4, D–E shows that TSA can change the refractory chromatin, and relieve the *HIST1* histone genes. This gives a molecular mechanistic insight into the low expression of *HIST1* histone genes in SCNT embryos. The *HIST1* histone genes have lain under an inactive chromatin state in SCNT blastocysts, and were relieved by TSA, which removes repressive chromatin remodelers and recruits various transcription activators to the locus. In support of this view, DNA methylation at *HIST1* histone gene promoters was reduced to levels comparable to those in IVF embryos after TSA treatment (Figure 4F). Therefore, our findings indicate that TSA, through modulation of the epigenetic state of the *HIST1* locus in SCNT blastocysts, renders the histone genes transcriptionally competent, which is important for proper development of the embryo (see below).

The SCNT blastocyst group expressed lower levels of histone genes compared with the IVF group. This is attributed to the reduced expression of *HIST1* histone genes, rather than other histone genes (Figure 3C). The four core histone molecules, H2A, H2B, H3, and H4, comprise a nucleosome, maintaining a ratio of one H3–H4 tetramer, and two H2A–H2B dimers. Unequal histone levels, such as weighing toward a particular molecule, might induce cell cycle arrest in SCNT embryos (Ghule *et al.* 2014). Since ∼100 histone-like genes with LOC-prefixed names in the cow genome are, as yet, uncharacterized, we were unable to estimate the stoichiometric balance among the four histone molecules from the blastocyst transcriptomes. Therefore, whether the reduced histone expression causes a hazardous imbalance and leads to cell cycle arrest in SCNT embryos, remains to be ascertained. Another hypothesis is that, despite the reduced levels, SCNT blastocysts are able to maintain stable and proportionate histone mRNA levels and, consequently, equilibrate among them. Irrespective of these scenarios, the premise of successful clonal development is that all histone genes, which reside scattered on the chromosomes, are coordinately expressed against genome-wide reprogramming processes.

The effect of reduced *HIST1* histone gene expression on SCNT embryos is unresolved. It is known that histone synthesis is tightly coupled to that of DNA, and, likewise, inhibition of the latter during the replication phase of the cell cycle causes rapid decline in the former (Plumb *et al.* 1983; Sittman *et al.* 1983; Su *et al.* 2004; Ghule *et al.* 2014). Therefore, if the reduced transcription affects protein level, the diminished amount of histones might cause a deregulated cell cycle progression (*e.g.*, delayed S-phase entry) in blastomeres. Unlike normal embryonic cell cycle, which involves rapid successions of DNA replication without the intermittent G1 and G2 phases, that of SCNT embryos is a lengthy process that can be observed in typical differentiated somatic cells (Becker *et al.* 2006; Boiani *et al.* 2003; Balbach *et al.* 2012). Consequently, SCNT blastocysts frequently suffer from low cell counts. If this is true, the reduced expression of *HIST1* histone genes is indicative of poor genome reprogramming, which must be overcome for normal clonal development.

Numerous and variable transcriptomic dissimilarities occur unpredictably between SCNT embryos, indicating the stochastic nature of reprogramming processes. Nevertheless, some changes in these embryos are nonrandom and consistent, represented by those in the *HIST1* locus. In addition, we recently reported a 400-kb locus harboring zinc-finger (ZNF) protein family genes in chromosome 18 that were coordinately downregulated in fSCNT blastocysts, showing a feature of reprogramming-resistant regions (Min *et al.* 2015). Therefore, it is not entirely true that each SCNT embryo is unique in terms of its gene expression profile. To the best of our knowledge, this is the first study to report the identification of a reprogramming-resistant megabase-stretch in the genome of bovine SCNT embryos. Extensive efforts to discover such regions, which persistently escape reprogramming, might help establish a reprogramming error-prone genomic map in SCNT embryos. Such a map will shed light on the mechanisms underlying local and global reprogramming events, and help improve the efficiency of such events.

## Supplementary Material

Supplemental Material

## References

[bib1] Albig W. Kioschis P. Poustka A. Meergans K. Doenecke D., 1997 Human histone gene organization: nonregular arrangement within a large cluster. Genomics 40(2): 314–322. 9119399 10.1006/geno.1996.4592

[bib2] Amano T. Tani T. Kato Y. Tsunoda Y., 2001 Mouse cloned from embryonic stem (ES) cells synchronized in metaphase with nocodazole. J. Exp. Zool. 289(2): 139–145. 11169501 10.1002/1097-010x(20010201)289:2<139::aid-jez7>3.0.co;2-6

[bib3] Balbach S. T. Esteves T. C. Houghton F. D. Siatkowski M. Pfeiffer M. J., 2012 Nuclear reprogramming: kinetics of cell cycle and metabolic progression as determinants of success. PLoS One 7(4): e35322. 22530006 10.1371/journal.pone.0035322 PMC3329427

[bib4] Becker K. A. Ghule P. N. Therrien J. A. Lian J. B. Stein J. L., 2006 Self-renewal of human embryonic stem cells is supported by a shortened G1 cell cycle phase. J. Cell. Physiol. 209(3): 883–893. 16972248 10.1002/jcp.20776

[bib5] Boiani M. Eckardt S. Leu N. A. Scholer H. R. McLaughlin K. J., 2003 Pluripotency deficit in clones overcome by clone-clone aggregation: epigenetic complementation? EMBO J. 22(19): 5304–5312. 14517267 10.1093/emboj/cdg507 PMC204490

[bib6] Cui X. S. Xu Y. N. Shen X. H. Zhang L. Q. Zhang J. B., 2011 Trichostatin A modulates apoptotic-related gene expression and improves embryo viability in cloned bovine embryos. Cell. Reprogram. 13(2): 179–189. 21473694 10.1089/cell.2010.0060

[bib7] Eggan K. Akutsu H. Loring J. Jackson-Grusby L. Klemm M., 2001 Hybrid vigor, fetal overgrowth, and viability of mice derived by nuclear cloning and tetraploid embryo complementation. Proc. Natl. Acad. Sci. USA 98(11): 6209–6214. 11331774 10.1073/pnas.101118898 PMC33447

[bib8] Eliassen K. A. Baldwin A. Sikorski E. M. Hurt M. M., 1998 Role for a YY1-binding element in replication-dependent mouse histone gene expression. Mol. Cell. Biol. 18(12): 7106–7118. 9819397 10.1128/mcb.18.12.7106 PMC109292

[bib9] Fletcher C. Heintz N. Roeder R. G., 1987 Purification and characterization of OTF-1, a transcription factor regulating cell cycle expression of a human histone H2b gene. Cell 51(5): 773–781. 3677172 10.1016/0092-8674(87)90100-0

[bib10] Ghule P. N. Xie R. L. Medina R. Colby J. L. Jones S. N., 2014 Fidelity of histone gene regulation is obligatory for genome replication and stability. Mol. Cell. Biol. 34(14): 2650–2659. 24797072 10.1128/MCB.01567-13 PMC4097655

[bib11] Hill J. R. Winger Q. A. Long C. R. Looney C. R. Thompson J. A., 2000 Development rates of male bovine nuclear transfer embryos derived from adult and fetal cells. Biol. Reprod. 62(5): 1135–1140. 10775159 10.1095/biolreprod62.5.1135

[bib12] Jee B. C. Jo J. W. Lee J. R. Suh C. S. Kim S. H., 2012 Effect of trichostatin A on fertilization and embryo development during extended culture of mouse oocyte. Zygote 20(1): 27–32. 21269543 10.1017/S0967199410000547

[bib13] Kang Y. K. Lee K. K. Han Y. M., 2003 Reprogramming DNA methylation in the preimplantation stage: peeping with Dolly’s eyes. Curr. Opin. Cell Biol. 15(3): 290–295. 12787770 10.1016/s0955-0674(03)00031-0

[bib14] Kishigami S. Mizutani E. Ohta H. Hikichi T. Thuan N. V., 2006 Significant improvement of mouse cloning technique by treatment with trichostatin A after somatic nuclear transfer. Biochem. Biophys. Res. Commun. 340(1): 183–189. 16356478 10.1016/j.bbrc.2005.11.164

[bib15] Kishigami S. Bui H. T. Wakayama S. Tokunaga K. Van Thuan N., 2007 Successful mouse cloning of an outbred strain by trichostatin A treatment after somatic nuclear transfer. J. Reprod. Dev. 53(1): 165–170. 17077581 10.1262/jrd.18098

[bib16] Kwon S. Jeong S. Park J. S. Kang Y. K., 2015 Quantifying difference in gene expression profile between bovine blastocysts derived by in vitro fertilization and somatic cell nuclear transfer. Gene Expr. Patterns. 19(1–2): 14–20 10.1016/j.gep.2015.05.005 26101995

[bib17] Lanza R. P. Cibelli J. B. Blackwell C. Cristofalo V. J. Francis M. K., 2000 Extension of cell life-span and telomere length in animals cloned from senescent somatic cells. Science 288(5466): 665–669. 10784448 10.1126/science.288.5466.665

[bib18] Ma T. Van Tine B. A. Wei Y. Garrett M. D. Nelson D., 2000 Cell cycle-regulated phosphorylation of p220(NPAT) by cyclin E/Cdk2 in Cajal bodies promotes histone gene transcription. Genes Dev. 14(18): 2298–2313. 10995387 10.1101/gad.829500 PMC316935

[bib19] Marzluff W. F. Duronio R. J., 2002 Histone mRNA expression: multiple levels of cell cycle regulation and important developmental consequences. Curr. Opin. Cell Biol. 14(6): 692–699. 12473341 10.1016/s0955-0674(02)00387-3

[bib20] Marzluff W. F. Gongidi P. Woods K. R. Jin J. Maltais L. J., 2002 The human and mouse replication-dependent histone genes. Genomics 80(5): 487–498. 12408966

[bib21] Min, B., S. Cho, J. S. Park, Y. G. Lee, N. Kim *et al.*, 2015 Transcriptomic features of bovine blastocysts derived by somatic cell nuclear transfer. G3 (Bethesda) 5(12): 2527–2538 10.1534/g3.115.020016 PMC4683625 26342001

[bib22] Ono Y. Shimozawa N. Ito M. Kono T., 2001 Cloned mice from fetal fibroblast cells arrested at metaphase by a serial nuclear transfer. Biol. Reprod. 64(1): 44–50. 11133657 10.1095/biolreprod64.1.44

[bib23] Park J. S. Jeong Y. S. Shin S. T. Lee K. K. Kang Y. K., 2007 Dynamic DNA methylation reprogramming: active demethylation and immediate remethylation in the male pronucleus of bovine zygotes. Dev. Dyn. 236(9): 2523–2533. 17676637 10.1002/dvdy.21278

[bib24] Plumb M. Stein J. Stein G., 1983 Influence of DNA synthesis inhibition on the coordinate expression of core human histone genes during S phase. Nucleic Acids Res. 11(22): 7927–7945. 6647036 10.1093/nar/11.22.7927 PMC326550

[bib25] Sittman D. B. Graves R. A. Marzluff W. F., 1983 Histone mRNA concentrations are regulated at the level of transcription and mRNA degradation. Proc. Natl. Acad. Sci. USA 80(7): 1849–1853. 6572946 10.1073/pnas.80.7.1849 PMC393707

[bib26] Su C. Gao G. Schneider S. Helt C. Weiss C., 2004 DNA damage induces downregulation of histone gene expression through the G1 checkpoint pathway. EMBO J. 23(5): 1133–1143. 14976556 10.1038/sj.emboj.7600120 PMC380976

[bib27] Wang Z. F. Krasikov T. Frey M. R. Wang J. Matera A. G., 1996 Characterization of the mouse histone gene cluster on chromosome 13: 45 histone genes in three patches spread over 1Mb. Genome Res. 6(8): 688–701. 8858344 10.1101/gr.6.8.688

[bib28] Whitfield M. L. Zheng L. X. Baldwin A. Ohta T. Hurt M. M., 2000 Stem-loop binding protein, the protein that binds the 3′ end of histone mRNA, is cell cycle regulated by both translational and posttranslational mechanisms. Mol. Cell. Biol. 20(12): 4188–4198. 10825184 10.1128/mcb.20.12.4188-4198.2000 PMC85788

[bib29] Yeo S. Jeong S. Kim J. Han J. S. Han Y. M., 2007 Characterization of DNA methylation change in stem cell marker genes during differentiation of human embryonic stem cells. Biochem. Biophys. Res. Commun. 359(3): 536–542. 17548060 10.1016/j.bbrc.2007.05.120

[bib30] Zhao J. Kennedy B. K. Lawrence B. D. Barbie D. A. Matera A. G., 2000 NPAT links cyclin E-Cdk2 to the regulation of replication-dependent histone gene transcription. Genes Dev. 14(18): 2283–2297. 10995386 PMC316937

