# Supplementary material for: The HIST1 Locus Escapes Reprogramming in Cloned Bovine Embryos
Source: G3 (Bethesda). 2016 Mar 11;6(5):1365–71. doi: 10.1534/g3.115.026666 (PMC4856087; doi:10.1534/g3.115.026666)
Supplement: Supplemental Material [file supp_6_5_1365__index.html]

The HIST1 Locus Escapes Reprogramming in Cloned Bovine Embryos — Supplemental Material 

# The *HIST1* Locus Escapes Reprogramming in Cloned Bovine Embryos

## Supplemental Material for Min *et al.*, 2016

**Files in this Data Supplement:**

- Figure S1 - *HIST1* downregulation in the donor cells. (.pdf, 225 KB)
- Figure S2 - Expression levels of histone gene expression regulators in individual blastocysts. (.pdf, 125 KB)
