# Supplementary material for: The HIST1 Locus Escapes Reprogramming in Cloned Bovine Embryos
Source: G3 (Bethesda). 2016 Mar 11;6(5):1365–71. doi: 10.1534/g3.115.026666 (PMC4856087; doi:10.1534/g3.115.026666)
Supplement: Supplemental Material [file supp_g3.115.026666_FigureS2.pdf]

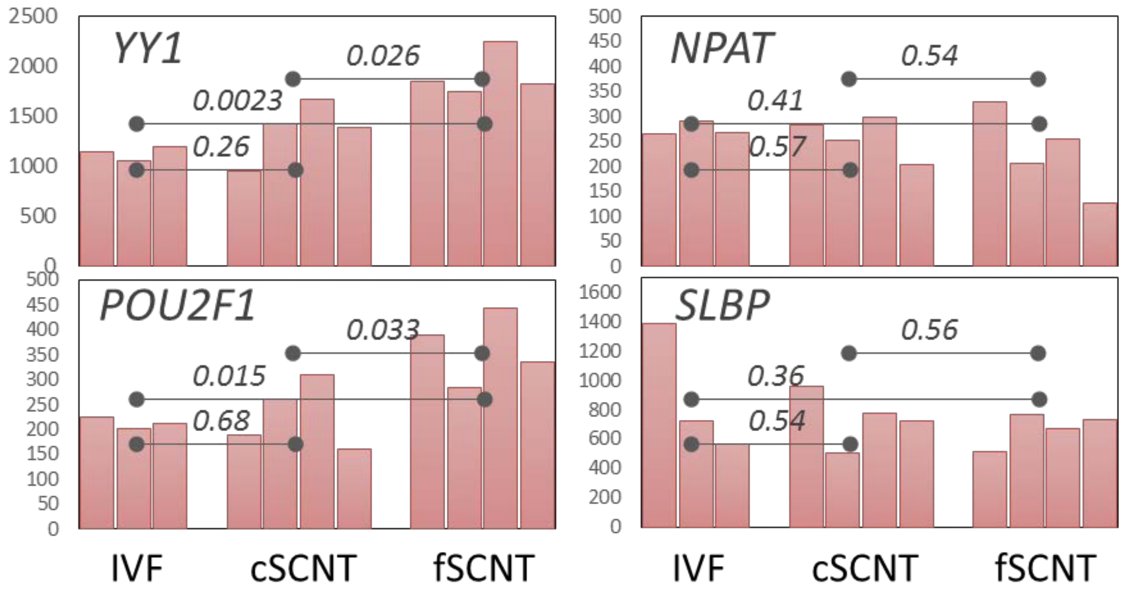

Figure S2. Expression levels of histone gene expression regulators in individual blastocysts

Expression levels of factors known to regulate coordinated histone gene expressions in each blastocyst were compared.
