# Supplementary material for: The HIST1 Locus Escapes Reprogramming in Cloned Bovine Embryos
Source: G3 (Bethesda). 2016 Mar 11;6(5):1365–71. doi: 10.1534/g3.115.026666 (PMC4856087; doi:10.1534/g3.115.026666)
Supplement: Supplemental Material [file supp_g3.115.026666_FigureS1.pdf]

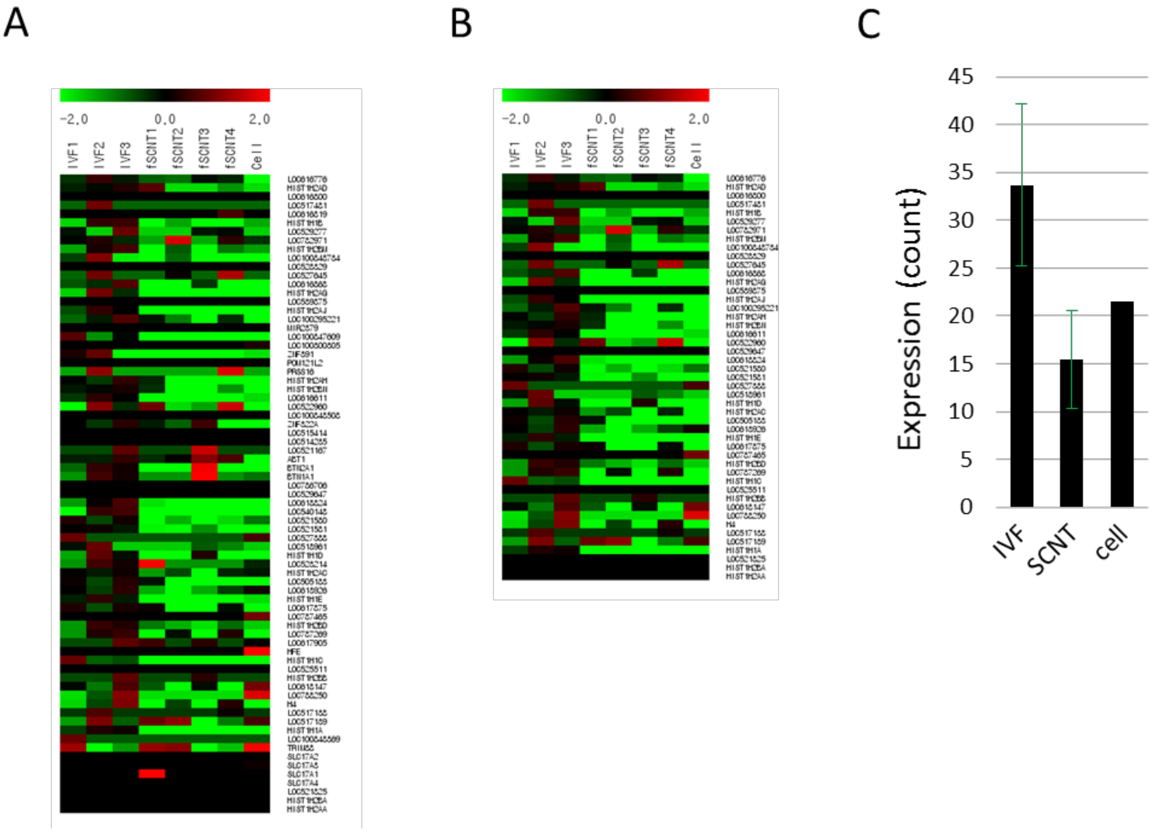

Figure S1. ***HIST1*** **downregulation in the donor cells**

A. Heatmap showing relative expression against IVF mean expression at *HIST1* locus in IVF, fSCNT, and the donor cell groups.

B. Heatmap showing relative expression against IVF mean expression of *HIST1* histone genes in IVF, fSCNT, and the donor cell groups.

C. Average expression levels of *HIST1* histone genes in IVF, fSCNT, and the donor cell groups. Error bars indicate standard deviations of replicates.
